# Physical therapy interventions for the correction of equinus foot deformity in post-stroke patients with triceps spasticity: A scoping review

**DOI:** 10.3389/fneur.2022.1026850

**Published:** 2022-10-28

**Authors:** Isabella Campanini, Maria Chiara Bò, Francesca Salsi, Maria Chiara Bassi, Benedetta Damiano, Sara Scaltriti, Mirco Lusuardi, Andrea Merlo

**Affiliations:** ^1^LAM – Motion Analysis Laboratory, Neuromotor and Rehabilitation Department, San Sebastiano Hospital, Azienda USL-IRCCS di Reggio Emilia, Correggio, Italy; ^2^Merlo Bioengineering, Parma, Italy; ^3^Medical Library, Azienda USL-IRCCS di Reggio Emilia, Reggio Emilia, Italy; ^4^Neuromotor and Rehabilitation Department, Azienda USL-IRCCS Reggio Emilia, Correggio, Italy

**Keywords:** equinus foot deformity, stroke, physical therapy, rehabilitation, spasticity

## Abstract

**Objective:**

Equinus foot deformity (EFD) is the most common deformity following a stroke. Several approaches have been suggested for its correction, including pharmacological, surgical, and physical therapy (PT) interventions. This scoping review aims to map and synthesize the available evidence focusing on physical therapy treatments for EFD caused by triceps surae (TS) spasticity.

**Methods:**

Scoping review methodological frameworks have been used. Pubmed, Cinahl, and Cochrane databases were searched for primary literature. Studies focusing on the treatment of EFD in adult stroke patients were included only when the intervention involved PT treatments and presented at least one outcome measure for the functional and/or structural condition of the TS. Data were systematically collected and reported in tables inclusive of type of intervention, sample characteristics, dosage, comparators, outcomes, follow-up timeline, and treatment efficacy. A narrative synthesis was also added.

**Results:**

Of the 642 experimental or observational screened studies, 53 were included, focusing on stretching exercises, shock waves, electrical stimulation, dry needling, TENS, vibration therapy, ultrasounds, cryotherapy, and active physiotherapy. Patients with EFD benefited from specific physical therapy treatments. These usually resulted in Modified Ashworth Scale reduction, typically by 1 point, and an increase in ROM. Interventions consisting of shock waves, dry needling, and electrostimulation showed the best results in reducing EFD. Heterogeneous dosage and delivery mode generally limited conclusions.

**Conclusions:**

This scoping review summarized available primary literature based on PT treatments for the correction of EFD. By highlighting the remaining gaps in knowledge, it provides a reference for future studies on this pathology. Further investigations are necessary to pinpoint the best dosage and delivery methods. Future studies should investigate whether early rehabilitation programs started during the acute phase might help prevent or limit the development of secondary deformities.

## Introduction

Equinus foot deformity (EFD) is the most common acquired deformity of the lower limb following a stroke. It is characterized by a downward deformity of the ankle, usually associated with an internal rotation of the foot, causing varus-supination. Sometimes, clawed toes are also present, further affecting the physiological anatomy of the foot (1).

EFD is the first cause of disability in stroke patients since it alters normal gait pattern causing pain and impairing ankle stability and passive dorsiflexion during the stance phase and limits foot clearance during the swing phase (2, 3). Given the need for orthotic devices, assistance during transfers, and the increased risk of falling (4), the patients' quality of life is negatively affected by this deformity and the possibilities of returning to a normal life are considerably reduced (5, 6).

Several factors can combine and cause EFD. Firstly, spasticity, co-contraction, spastic dystonia, and paresis cause an imbalance between the dorsiflexor and plantarflexor muscles, and between the invertor and evertor muscles. Many lower limb muscles can cause equinus or supination deformities if their activity is altered and not counterbalanced by the antagonistic muscle groups, as clearly illustrated by Campanini et al. (1). Moreover, changes in soft tissues such as increased stiffness, viscosity, and contractures contribute to altering the effector peripheral system, influencing central responses during movement (1, 7).

Spasticity has been treated in several ways, including surgical, pharmacological, PT, and orthotic interventions (8–10). Physiotherapy represents a substantial component of the non-invasive approaches used in managing post-stroke spasticity and, for this reason it is one of the first recommended treatments. Many PT modalities have been described in literature, with new interventions being introduced over the years aiming to correct EFD. Given the heterogeneity of those treatments, the modalities of their delivery, and the different rationale on which they are based, as of now, it would be premature to conduct a systematic review that synthesizes the results obtained from the studies by considering them as uniform. Scoping reviews are employed when summarizing what already exists in a vast and still developing field, highlighting any methodological gaps that need to be filled, and directing further focused studies (11–13). In line with our aims, a scoping review design was chosen for this current study.

We were interested in mapping current available evidence on PT treatments of EFD in adult stroke patients. For each intervention, we focused on the length of each treatment and the characteristics of the included population; we identified the strengths and limitations of current clinical practices, thus helping rehabilitation professionals to employ this knowhow into their everyday practice by collecting and summarizing the results and leading the way for future investigations.

## Methods

According to the scoping review guidelines—an extension of the PRISMA guidelines for systematic reviews (14), we developed our study following a six-stage iterative process (12).

### Stage 1: Identifying the research question

The leading questions of our investigation were: (I) What are the physiotherapeutic interventions for EFD reduction caused by triceps surae (TS) spasticity? (II) Which type of stroke patients are these treatments delivered to (e.g., chronic, acute)? (III) Are there optimal dosages shared in literature? (IV) What outcomes do the treatments affect and what effects do they have?

### Stage 2: Identifying relevant studies

The search strategy was developed starting May 2021 by two researchers and a scientific librarian. Medline, Cinahl, and Cochrane databases were searched.

Searched keywords included: “equinus deformity,” “stroke,” “physiotherapy,” “rehabilitation,” “extracorporeal shock waves,” “dry needling,” “stretching,” “ultrasound,” “vibration,” “tens,” “electric stimulation,” “muscle spasticity,” “spastic paresis.” Medical Subjects Headings (MeSH) were included, when available, to ensure consistency of the search terms (see Supplementary material for the whole search strategy).

To avoid missing out on potentially relevant papers, we extended our search to include references of all the studies previously retrieved from the electronic databases. All titles and abstracts from these bibliographies were systematically screened and relevant papers were added to the list of the included studies.

### Stage 3: Selecting studies

#### Eligibility criteria

Studies had to include adult patients suffering from EFD and TS spasticity due to stroke. Studies focusing on the treatment of EFD were considered eligible if the intervention involved physiotherapy treatments and presented at least one outcome measure of the TS functional and/or structural condition. Articles were only eligible if full text was available in English and no time limitations were set. Studies were excluded if the samples included other neurological diseases responsible for muscle spasticity, such as cerebral palsy, multiple sclerosis, traumatic brain injuries, or spinal cord injuries. We also excluded studies involving patients affected by drop foot due to dorsiflexor paresis only (i.e., with no increase of the tonic stretch reflex in the plantarflexor muscles). Studies involving treatments of the upper limbs, or for lower limb muscles other than the plantarflexors, were excluded too. Studies using surgical or pharmacological treatments—such as surgical lengthening or botulinum neurotoxin injections—or alternative medicine treatments were not considered eligible. Papers were excluded if authors only recorded functional outcomes (e.g., walking ability), without any measurement directly related to TS impairment (either clinical or instrumental measurements of spasticity).

#### Types of studies

Scoping reviews have a flexible study design, that allows for the inclusion of any type of study the authors might consider suitable to answer their research questions (11). We included any papers with primary study designs, such as experimental or observational ones, including any relevant case-report.

#### Population

The population of interest involved adult (age ≥ 18y) stroke patients with EFD due to TS spasticity.

#### Context

No limitations were set regarding the context of the studies.

### Stage 4: Charting the data

Both researchers evaluated all relevant titles and abstracts independently, according to the inclusion and exclusion criteria. A third researcher was called upon to solve any discrepancy. When necessary, changes to the tables were made to improve the accuracy of the review.

According to PRISMA guidelines, scoping reviews do not require critical appraisals and quantitative synthesis of the included studies (11).

### Stage 5: Summarizing and reporting the data

Relevant data were collected and inserted in a Microsoft Excel database by author, year of publication, sample information, comparators, intervention types and dosages (timing, frequency, and intensity), outcome measures, statistical methodologies used for comparisons within and between groups, and the main results. Findings were then summarized in tables according to the intervention used and a narrative synthesis was also included.

### Stage 6: Consultation

During the entire process, we also reached out to neurological rehabilitation experts for advice in setting eligibility criteria and to help us retrieve any relevant studies that could have been missed by the search.

## Results

**Figure 1:**
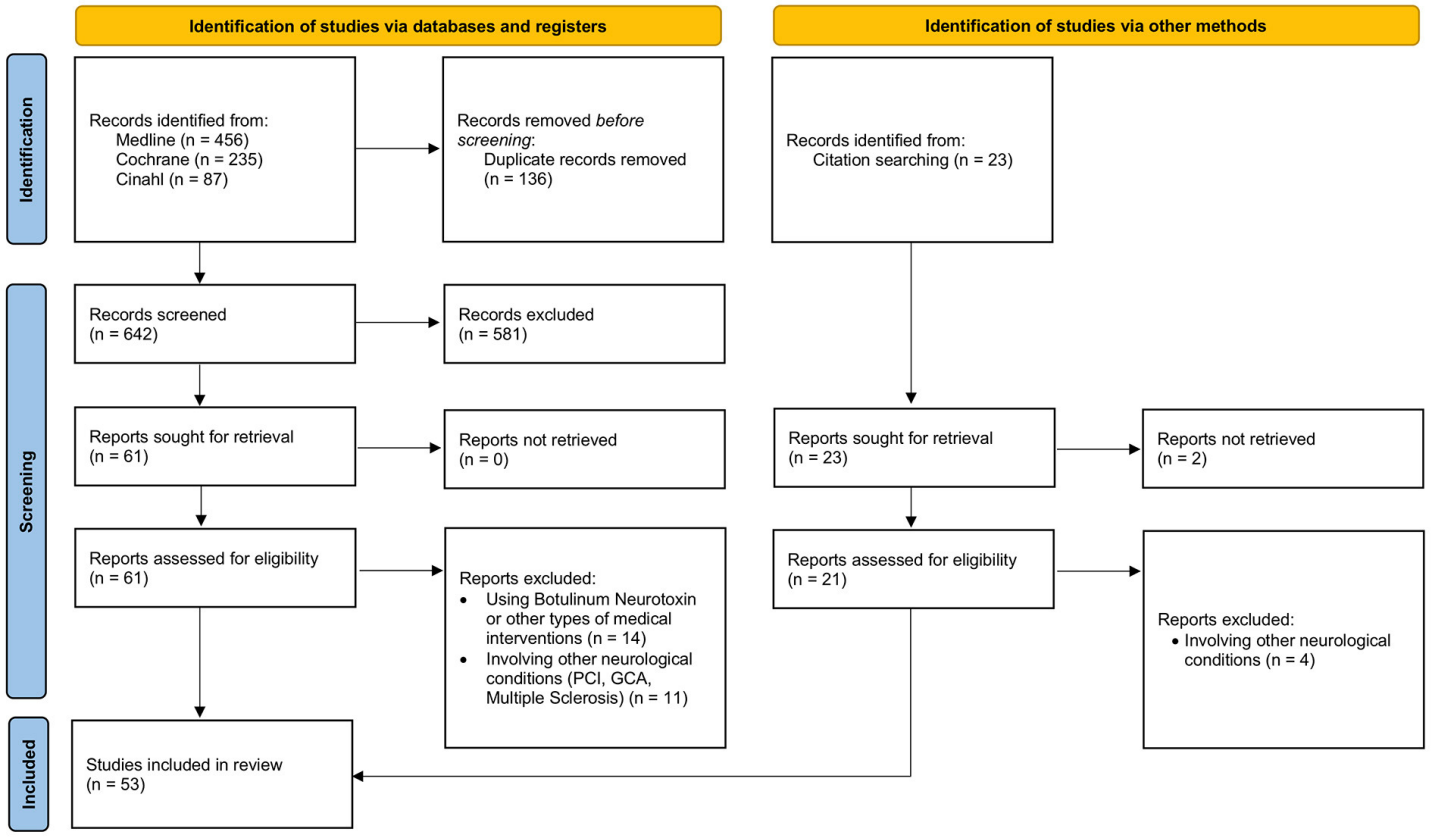
Flow chart of the literature search on physical therapy interventions for the correction of equinus foot deformity in post-stroke patients with triceps spasticity.

The search led to the identification of 778 articles. After the removal of duplicates, 642 records were screened, and 61 papers were selected for full-text screening. Of these, 36 were included in the scoping review, in addition to 17 studies identified manually, for a total of 53 articles. The flow chart in Figure 1 shows the article selection procedure, according to PRISMA guidelines (14).

The papers were published between 2001 and 2021, with samples ranging from 1 to 83 adult patients suffering from TS spasticity following a stroke. Thirty of the included studies were RCTs; the others had study designs without randomization or controls.

### Stretching

Twelve papers focused on stretching techniques for the treatment of EFD after stroke and these were also included in this review. These can be found in Table 1.

**Table 1:** Characteristics of studies using stretching for the treatment of triceps spasticity after stroke.

**Author**	**Sample**	**Intervention and dosage**	**Comparator**	**Outcomes**	**Follow-up** **(months)**	**Within group** **difference (over** **time)**	**Between group** **difference after** **treatment**
Pradines et al. (15) (RCT)	23 (12/11 IG/CG) patients with spasticity for at least 1 year (Tardieu angle ≥5°)	Usual care three times a week + 15-min stretching Once a day, for 1 year (self-managed)	CG: Usual care three times a week, for 1 year	Tardieu Angle, Fascicle Length (FL), Muscle Thickness (MT), 10 mAS	12	IG: Tardieu Angle (soleus, gastrocnemius) ↓, soleus FL, gastrocnemius FL, soleus MT, 10 mAS ↑, gastrocnemius MT ↔ CG: Tardieu Angle, FL, MT, 10 mAS ↔	Tardieu Angle, FL, soleus MT, 10 mAS^†^
Ghasemi et al. (16) (RCT)	45 (30/15 IG/CG) patients with spasticity for at least 3 months (MMAS≥1)	3-min functional stretching Three times a week, for 1 month	CG: Usual care	MMAS, Achilles Tendon Reflex (ATR) excitability, presence of clonus, pROM, H-reflex latency, H/M ratio, PA, MT, FL	2	IG: MMAS, ATR excitability, H-reflex latency, H/M ratio, PA, MT, FL ↔ Clonus ↓ pROM ↑ CG: MMAS, ATR excitability, clonus, pROM, H-reflex latency, H/M ratio, PA, MT, FL ↔	No statistical difference
Pradines et al. (17)	27 patients with spasticity (TS ≥ 5°)	15-min stretching Once a day, for 1 year (self-managed)	n.a.	Tardieu Angle Coefficient of shortening (Csh), 10 maS	12, 24, 36	Tardieu Angle, Csh ↓ 10 maS ↑	n.a.
Gao et al. (18)	10 patients with spasticity	60-min stretching (12 repetitions lasting 5 min each) with smart devices One single session	CG: 10 healthy subjects	Torque-angle ratio (stiffness) Achilles tendon length	n.a.	IG: Stiffness ↓ Achilles tendon length ↔	n.a.
Bakheit et al. (19) (RCT)	66 (22/22/22 IG1/IG2/IG3) patients with spasticity for at least 6 weeks (MAS>1)	IG1: 20-min isotonic stretching (with weight bearing) IG2: 20-min stretching (without weight bearing) IG3: 20-min isokinetic stretching One single session with smart devices	CG: 21 healthy subjects	H/M ratio H-reflex latency	One day	IG1, IG2, IG3: H/M ratio, H-reflex latency ↔ CG: H/M ratio, H-reflex latency ↔	No statistical difference
Chung et al. (20)	12 patients with chronic spasticity (MAS>1)	30-min stretching with smart devices One single session	CG: 10 healthy subjects	Peak reflex torque, ATR threshold Torque-angle ratio (stiffness), pROM, MVC	n.a.	IG: Peak reflex torque, stiffness ↓ ATR threshold ↔ pROM, MVC ↑ CG: Peak reflex torque, ATR threshold, pROM, stiffness, MVC ↔	n.a.
Maynard et al. (21)	66 (22/22/22 IG1/IG2/IG3) patients with spasticity for at least 6 weeks (MAS > 1)	IG1: 20-min isotonic stretching (with weight bearing) IG2: 20-min stretching (without weight bearing) IG3: 20-min isokinetic stretching One single session with smart devices	CG: 21 healthy subjects	Kinematic, dynamic and spatio-temporal parameters through gait analysis	One day	IG1, IG2, IG3: all parameters ↔ CG: all parameters ↔	No statistical difference
Selles et al. (22)	10 patients with spasticity (MAS ≥ 1)	45-min stretching with smart devices 3 times a week, for 1 month	n.a.	Torque-angle ratio (stiffness) pROM, aROM, MVC, ATR excitability, 10 mAS, VAS	n.a.	Stiffness ↓ pROM, MVC, 10 mAS, VAS ↑ Viscosity, aROM, ATR excitability ↔	n.a.
Yeh et al. (23)	30 patients with spasticity for at least 6 months (MAS > 1)	IG1: 30-min constant-angle stretching with smart devices One single session	IG2: 30-min constant-torque stretching with smart device, one single session after 1 week	MAS pROM, Reactive Torque	n.a.	IG1: MAS, RT ↓ pROM ↑ IG2: MAS, RT ↓ pROM ↑	RT^§^
Bressel et al. (24)	10 patients with spasticity for at least 3 months (MAS ≥ 1)	IG1: 30-min static stretching with smart devices One single session	IG2: 30-min cycling stretching, one single session after 1 week	Ankle joint angle and passive torque (stiffness) EMG activity, 10 MWT	n.a.	IG: Stiffness ↓ 10MWT ↔ IG2: Stiffness ↓ 10MWT ↔	No statistical difference
Zhang et al. (25)	4 patients with chronic spasticity	Stretching with smart devices One single session	n.a.	Torque-angle ratio (stiffness) aROM, pROM, ATR excitability	n.a.	Stiffness, viscosity, ATR excitability ↓ aROM, pROM ↑	n.a.
Tsai et al. (26)	17 patients with spasticity (1 ≤ MAS ≤ 4)	30-min stretching on a tilt-table One single session	n.a.	MAS pROM, H/M ratio, F/M ratio	n.a.	MAS ↔ pROM, F/M ratio ↑ H/M ratio ↓	n.a.

↑ Statistically significant increase.

↓ Statistically significant decrease.

↔ No statistical difference.

† IG overcomes comparator.

§ Comparator overcomes IG.

IG, Intervention Group; CG, Control Group; n.a. not available; RCT, Randomized Controlled Trial; 10 mAS, 10-meter Ambulation Scale; FL, Fascicle Length; MT, Muscle Thickness; MMAS, Modified Modified Ashworth Scale; pROM, passive Range of Motion; ATR, Achilles Tendon Reflex; PA, Pennation Angle; Csh, Coefficient of Shortening; MAS, Modified Ashworth Scale; aRoM, active Range of Motion; MVC, Maximal Voluntary Contraction; VAS, Visual Analog Scale; RT, Reactive Torque; EMG, Electromyography; 10 MWT, 10-meter Walking Test.

The intervention groups were offered various types of stretching exercises (isotonic/isokinetic, with/without weight-bearing, constant-angle/constant-torque, static/cyclic) and in 75% of the cases the treatment was provided with the aid of smart devices. In the remaining three studies, treatments were delivered by physiotherapists or self-managed by patients. Eight studies included a control group: four used a sample of healthy individuals, two studies compared different types of stretching exercises, and two others used usual care as a comparator.

Dosage was highly heterogeneous among studies, from 3 to 60 min per session, from three times per week to daily workouts, and from single sessions to year-long interventions. The parameters taken into consideration were: treatment intensity per single session, duration of each session, frequency of sessions per week, and/or overall duration of the intervention.

Six out of eight studies that proposed a single stretching session registered a reduction in stiffness and in the Modified Ashworth Scale (MAS) scores or an increase in ankle joint mobility immediately after the treatment, whereas the H/M ratio did not show any significant changes (18–21, 23–26). Two studies offered a month-long stretching session, three times a week: Ghasemi observed a significant improvement only in the pennation angle and muscle thickness after 3 min of stretching (16), while Selles observed a reduction in stiffness and an increase in ROM, strength, and gait speed in 10 patients who received 45 min-long stretching sessions (22). Finally, Pradines conducted a prospective study and an RCT in which patients performed a self-administered daily stretching program for 1 year and recorded a reduction in spasticity on the Tardieu Angle. This reduction differed from the control group by 4 degrees at the soleus and by 7 degrees at the gastrocnemius. An increase in walking speed was also found (15). It is worth mentioning that these results were maintained at the 2- and 3-year follow-ups (17).

### Shock waves

Shock waves are high amplitude waves with fast pressure changes that have been extensively employed for the spasticity caused by stroke or other neurological conditions in the treatment of EFD. Nine studies using this technique were included in this scoping review, the specifics of which are shown in Table 2. Treatments were delivered by MDs as physiatrists in six out of nine studies and by physiotherapists in the remaining three studies.

**Table 2:** Characteristics of studies using shock waves for the treatment of triceps spasticity after stroke.

**Author**	**Sample**	**Intervention and dosage**	**Comparator**	**Outcomes**	**Follow-up** **(months)**	**Within group** **difference (over time)**	**Between group** **difference after** **treatment**
Lee et al. (27) (RCT)	18(9/9 IG/CG) patients with spasticity for at least 3 months (MAS > 1)	ESWT (2,000 shots, 0.1 mJ/mm^2^) in the medial gastrocnemius + physical therapy One single session	CG: Sham stimulation + physical therapy	MAS pROM, FMA, Achilles Tendon Length (ATL), Fascicle Length (FL), Muscle Thickness (MT), Pennation Angle (PA)	1	IG: MAS, pROM ↔ FMA, FL ↑ ATL, MT, PA ↓ CG: MAS, pROM, ATL, MT, PA, FL ↔ FMA ↑	ATL, FL, MT, PA^†^
Radinmehr et al. (28) (RCT)	32 (16/16 IG1/IG2) patients with chronic spasticity (MMAS > 1)	IG1: rSWT (2,000 shots, 0.34 mJ/mm^2^) in the gastrocnemius One single session	IG2: Ultrasound therapy	MMAS aROM, pROM, TUG, H-reflex latency, PPFT	n.a.	IG1: MMAS, TUG, PPFT ↓ AROM, pROM, H-reflex latency ↑ IG2: MMAS, TUG, PPFT ↓ AROM, pROM, H-reflex latency ↑	No statistical difference
Wu et al. (29) (RCT)	32 (16/16 IG1/IG2) patients with spasticity for at least 6 months (MAS > 1)	IG1: rSWT (1,500 shots, 0.1 mJ/mm^2^) in the gastrocnemius and in the soleus Once a week, for 3 weeks	IG2: fSWT (1,500 shots, 2.0 bar) in the gastrocnemius and in the soleus	MAS, Tardieu Angle pROM, 10 MWT, Dynamic Foot Contact Area (DFCA)	2	IG1: MAS, Tardieu Angle ↓ PROM, DFCA ↑ 10MWT ↔ IG2: MAS, Tardieu Angle ↓ pROM, DFCA, DFCA, 10MWT ↔	PROM, DFCA^†^
Sawan et al. (30)	40 (20/20 IG/CG) patients with chronic spasticity (MAS > 1)	ESWT (1,500 shots) in the gastrocnemius + physical therapy Once a week, for 6 weeks	CG: Sham stimulation + physical therapy	H/M ratio aROM, 10 MWT	n.a.	IG: H/M ratio, 10 MWT ↓ aROM ↑ CG: H/M ratio, 10 MWT ↓ aROM ↑	H/M ratio, aROM^†^
Taheri et al. (31) (RCT)	25 (13/12 IG/CG) patients with spasticity from 1 month (MAS > 1)	fSWT (1,500 shots, 0.1 mJ/mm^2^) in the gastrocnemius + stretching exercises Once a week, for 3 weeks	CG: Stretching exercises	MAS VAS, pROM, Clonus Score (CS), 3 MWT, LEFS	3	IG: MAS, VAS ↓ pROM, LEFS ↑ CS, 3 MWT ↔ CG: MAS, VAS, pROM, LEFS, CS ↔ 3 MWT ↓	MAS, pROM, LEFS^†^
Radinmehr et al. (32)	12 patients with spasticity from at least 1 month (MAS > 1)	rSWT (2,000 shots, 0.34 mJ/mm^2^) in the gastrocnemius One single session	n.a.	MMAS aROM, pROM, TUG, H-reflex, H/M ratio, PPFT	n.a.	MMAS, PPFT, TUG ↓ aROM, pROM, H-reflex ↑ H/M ratio ↔	n.a.
Santamato et al. (33)	23 patients with spasticity for at least 5 months (MAS > 1)	fSWT (1,500 shots, 0.1 mJ/mm^2^) in the gastrocnemius and in the soleus One single session	n.a.	MAS pROM, Tibial Nerve Conduction (TNC), F-wave latency	1	MAS ↓ pROM ↑ (but maintained at 1 month only in patients with Heckmatt 1–3) TNC, F-wave latency ↔	n.a.
Moon et al. (34)	30 patients with spasticity for at least 1 month (MAS > 1)	One sham fSWT session physical therapy + fSWT (1,500 shots, 0.089 mJ/mm^2^) in the gastrocnemius Once a week, for 3 weeks	n.a.	MAS Clonus Score (CS), pROM, FMA, Peak Eccentric Torque (PET), Torque Threshold Angles (TTAs)	1	MAS, PET ↓ TTAs ↑ (But non-significant at follow-up at 1 month) CS, pROM, FMA ↔	n.a.
Sohn et al. (35)	10 patients with spasticity for at least 15 months (MAS > 1)	fSWT (1,500 shots, 0.1 mJ/mm^2^) in the gastrocnemius One single session	CG: 10 healthy subjects who underwent the same treatment	MAS F-waves latency, H-reflex latency, H/M ratio, Tibial Nerve Conduction (TNC)	n.a.	IG: MAS ↓ F-wave latency, H-reflex latency, H/M ratio, TNC ↔ CG: MAS not assessed F-wave latency, H-reflex latency, H/M ratio, TNC ↔	n.a.

↑ Statistically significant increase.

↓ Statistically significant decrease.

↔ No statistical difference.

† IG overcomes comparator.

IG, Intervention Group; CG, Control Group; n.a. not available; RCT, Randomized Controlled Trial; MAS, Modified Ashworth Scale; ESWT, External Shock-Wave Therapy; aROM, active Range of Motion; FMA, Fugl-Meyer Assessment; ATL, Achilles Tendon Length; FL, Fascicle Length; MT, Muscle Thickness; PA, Pennation Angle; rSWT, radial Shock-Wave Therapy; MMAS, Modified Modified Ashworth Scale; pROM, passive Range of Motion; TUG, Timed Up and Go test; PPFT, Passive Plantarflexor Torque; fSWT, focused Shock-Wave Therapy; 10MWT 10-meter Walking Test; DFCA, Dynamic Foot Contact Area; VAS, Visual Analog Scale; CS, Clonus Score; 3MWT, 3-meter Walking Test; LEFS, Lower Extremity Functional Score; TNC, Tibial Nerve Conduction; PET, Peak Eccentric Torque; TTAs, Torque Threshold Angles.

Shock waves were delivered in radial or focal mode, which differs according to the surface area being covered: the most frequent dosage was between 1,500 and 2,000 shots, 0.1–0.3 mJ/mm^2^, with 55% of the authors suggesting a single session while the remaining authors offered 3–6 week-long treatments.

Control groups included healthy subjects, other types of physical therapy, or sham shock waves delivered while keeping the device off. Eighty-seven percent of the trials obtained a reduction in MAS values, with an average reduction in MAS score of about 1. Similar results were at times recorded at the one-and three-month follow-ups. The same percentage of studies registered an increase in active and passive ankle ROM values (2–15-degree increase), even over the long term.

### Electrical stimulation

Electrical stimulation uses current that can be applied to muscles, promoting fiber contraction. Seven studies investigated its effects on EFD with triceps spasticity in stroke patients. Physiotherapists were involved in the delivery of treatments in all the included studies. The papers included in this scoping review are summarized in Table 3.

**Table 3:** Characteristics of studies using electrical stimulation for the treatment of triceps spasticity after stroke.

**Author**	**Sample**	**Intervention and dosage**	**Comparator**	**Outcomes**	**Follow-up** **(months)**	**Within group** **difference (over** **time)**	**Between group** **difference after** **treatment**
Ganesh et al. (36) (RCT)	83 (27/30/26 IG1/IG2/CG) patients with spasticity for at least 3 months (MCSS ≥ 7)	IG1: 80-min rehabilitation (with task-oriented exercises) + 10-min Faradic Current (100 Hz) on peroneal and tibial nerves IG2: 80-min rehabilitation (with task-oriented exercises) + 10-min Russian Current (2,500 Hz) on peroneal and tibial nerves Five days a week, for 6 weeks	CG: 80-min rehabilitation (with task-oriented exercises)	MMAS aROM, pROM, mEFAP	n.a.	IG1, IG2: MMAS, mEFAP ↓ aROM, pROM ↑ CG: MMAS, mEFAP ↓ aROM, pROM ↑	MMAS, pROM^†^ (IG2) (effect sizes reported only)
Yang et al. (37) (RCT)	25 (8/9/8 IG1/IG2/CG) with spasticity for at least 6 months	IG1: 20-min NMES (50 Hz) on tibialis anterior + 15-min gait training IG2: 20-min NMES (50 Hz) on medial gastrocnemius + 15-min gait training Three days a week, for 7 weeks	CG: 20-min stretching and ROM exercises + 15-min gait training	MAS Gastrocnemius EMG-activity, aROM, strength, gait analysis parameters	n.a.	IG1: MAS ↓ aROM, strength ↔ IG2: MAS, aROM, strength ↔ CG: MAS, aROM, strength ↔	Strength^†^ (IG1)
Sharif et al. (38) (RCT)	38 (19/19 IG/CG) patients with sub-acute spasticity	30-min FES (40 Hz) on tibialis anterior + usual rehabilitation program Five days a week, for 6 weeks	CG: 10-min electrical stimulation on tibialis anterior + usual rehabilitation program	MAS FMA, BBS, TUG, Gait Dynamic Index (GDI)	n.a.	IG: MAS, TUG ↓ FMA, BBS, GDI ↑ CG: MAS, FMA, BBS ↑ TUG, GDI ↓	MAS, FMA, TUG, BBS, GDI^†^
Suh et al. (39) (RCT)	42 (21/21 IG/CG) patients with spasticity for at least 6 months (MAS ≥ 2)	30-min Bobath approach + 60-min Interferential Current (100 Hz) on the gastrocnemius One single session	CG: 30-min Bobath approach + sham Interferential Current	MAS Functional Reach Test (FRT), BBS, TUG, 10 MWT	n.a.	IG: MAS, TUG, 10 MWT↓ FRT, BBS ↑ CG: MAS, TUG, 10 MWT ↓ FRT, BBS ↔	MAS, FRT, BBS, TUG, 10 MWT^†^
Sabut et al. (40)	51 (27/24 IG/CG) patients with spasticity for at least 3 months	1-h rehabilitation program + 30-min electrical stimulation (35 Hz) on tibialis anterior Five days a week, for 3 months	CG: 1-h rehabilitation program, 5 days a week, for 3 months	MAS MRC, aROM, pROM, FMA	n.a.	IG: MAS ↓ MRC, aROM, pROM, FMA ↑ CG: MAS ↓ MRC, aROM, pROM, FMA ↑	MAS, MRC, aROM, pROM, FMA^†^
Bakhtiary et al. (41) (RCT)	35 (17/18 IG/CG) patients with spasticity	10-min infrared + 15-min Bobath approach + 9-min Faradic Current (100 Hz) on the tibialis anterior 20 daily sessions	CG: 10-min infrared + 15-min Bobath approach	MAS pROM, MRC, H/M ratio	n.a.	IG: MAS, H/M ↓ pROM ↑ MRC ↔ CG: MAS, H/M ↓ pROM ↑ MRC ↔	No statistical difference
Chen et al. (42)	24 (12/12 IG/CG) patients with spasticity for at least 1 year (MAS > 1)	20-min electrical stimulation (20 Hz) on the gastrocnemius Six days a week, for 1 month	CG: Sham electrical stimulation	MAS F/M ratio, H-reflex latency, 10-mAS	n.a.	IG: MAS, F/M ratio, 10-mAS ↓ H-reflex latency ↑ CG: MAS, F/M ratio, H-reflex latency, 10-mAS ↔	Not computed

↑ Statistically significant increase.

↓ Statistically significant decrease.

↔ No statistical difference.

† IG overcomes comparator (listed in brackets when multiple).

IG, Intervention Group; CG, Control Group; n.a. not available; RCT, Randomized Controlled Trial; MCSS, Modified Composite Spasticity Score; MMAS, Modified Modified Ashworth Scale; aROM, active Range of Motion; pROM, passive Range of Motion; mEFAP, modified Emory Functional Ambulation Profile; NMES, Neuromuscular Electrical Stimulation; MAS, Modified Ashworth Scale; EMG, Electromyography; FES, Functional Electrostimulation; TUG, Timed Up and Go; FMA, Fugl-Meyer Assessment; BBS, Berg Balance Scale; GDI, Gait Dynamic Index; 10MWT, 10-meter Walking Test; FRT, Functional Reach Test; MRC, Medical Research Council; 10-mAS, 10-m Ambulation Speed.

Several types of current were used, including Faradic (consisting of a double phase, the first is a low intensity and long duration current, the second is a high intensity and short duration one), Russian (an alternating current with a 2.5 kHz frequency that is burst modulated at a 50 Hz frequency with a 50% duty cycle), and Interferential (produced by the interference of two medium-frequency sinusoidal alternating currents of slightly different frequencies). Moreover, functional electric stimulation (FES) was also employed. Dosage parameters varied by modality, and the current was applied to both plantarflexor and dorsiflexor muscles. Only one study measured the effects of electrical stimulation immediately after the single session treatment (39), while the others created month-long programs, with treatments occurring ~5 times per week. All authors included a control group, which was treated with infrared, targeted PT exercises, or placebo stimulation. One study compared FES and traditional electrical stimulation to the tibialis anterior in addition to the usual rehabilitation protocol (38). No study performed any follow-ups and efficacy was assessed right after the end of the treatment.

The MAS score was significantly decreased on average by 1 point in all groups. Ganesh found the Faradic current performed better than the Russian current in reducing spasticity (36). Sharif also recorded a greater improvement in spasticity outcomes after FES rather than with traditional electrical stimulation (38). Passive ROM improved about 13 degrees above baseline values, particularly when using Russian current treatments (36) or electrical stimulation combined with physical therapy (40). Gait speed increased when compared to the performance of groups who had received placebo stimulations (39, 42), whereas it did not reach statistical significance when comparing those who performed 80 min of task-oriented exercise rehabilitation (36).

### Dry needling

Dry needling is a novel technique used mainly in the management of orthopedic pathologies. When needles prick the tissues, there is a neural, connective, muscular, and blood flow stimulation (43, 44). Moreover, it supposedly disrupts contracted cytoskeletal structures, thus reducing muscle stiffness (45). Recently, it has also been introduced for the treatment of neurological issues. Six studies were retrieved where it was used as an intervention on EFD with TS spasticity following stroke (see the characteristics in Table 4). Treatments were delivered by physiotherapists in four out of six studies.

**Table 4:** Characteristics of studies using dry needling for the treatment of triceps spasticity after stroke.

**Author**	**Sample**	**Intervention and dosage**	**Comparator**	**Outcomes**	**Follow-up** **(months)**	**Within group** **difference (over time)**	**Between group** **difference after** **treatment**
Ghannadi et al. (43) (RCT)	24 (12/12 IG/CG) patients with spasticity for at least 6 months (MMAS > 1)	Dry needling with fast-in and fast-out technique in the gastrocnemius 3 sessions in a week	CG: Sham dry needling with blunt needle	MMAS TUG, Single Leg Stance (SLS), 10 MWT, Barthel Index (BI), aROM, pROM, Pennation Angle (PA), Muscle Thickness (MT)	1	IG: MMAS, TUG, 10 MWT, PA, MT ↓ pROM, SLS, BI ↑ aROM ↔ CG: MMAS, TUG, 10 MWT, PA, MT, pROM, aROM, SLS, BI ↔	MMAS, TUG, SLS, 10 MWT, BI, pROM, PA, MT^†^
Hadi et al. (46)	6 patients with spasticity for at least 6 months (MMAS ≥ 1)	Dry needling in the gastrocnemii and in the soleus One single session	n.a.	MMAS TUG, Muscle Thickness (MT), Pennation Angle (PA), Fascicle Length (FL)	n.a.	MMAS, TUG, MT, PA ↓ FL ↑	n.a.
Sànchez-Mila et al. (47) (RCT)	26 (14/12 IG/CG) patients with spasticity	Dry needling with fast-in and fast-out technique in the tibialis posterior + Bobath approach One single session	CG: Bobath approach	MMAS FMA, LoS test	n.a.	IG: MMAS ↓ FMA^Balance^, FMA^Sensory^, FMA^RoM^ ↑ FMA^LowEx^, FMA^JointPain^, LoS test ↔ CG: MMAS ↓ FMA, LoS test ↔	MMAS, FMA^Balance, Sensory, RoM†^
Calvo et al. (44)	1 patient with spasticity for 30 months	Dry needling in the gastrocnemius One single session	n.a.	Maximal radial muscle displacement	3	Maximal radial muscle displacement ↑	n.a.
Salom-Moreno et al. (45) (RCT)	34 (17/17 IG/CG) patients with spasticity	Dry needling with fast-in and fast-out techniques in the gastrocnemius and tibialis anterior One single session	CG: No intervention	MMAS Pressure Pain Thresholds (PPT), Baropodometric values	n.a.	IG: MMAS ↓ PPT ↑ CG: MMAS, PPT ↔	MMAS, PPT^†^
Fink et al. (48) (RCT)	25 (13/12 IG/CG) with spasticity for at least 7 months	Dry needling in acupuncture points Two times a week, for 1 month	CG: Sham needles placed in non-acupoints	MAS aROM, pROM, 2 MWT, H-reflex, H/M ratio	n.a.	IG: MAS, aROM, pROM, 2MWT ↔ H/M ratio ↓ CG: MAS, aROM, pROM, 2MWT ↔ H/M ratio ↓	H/M ratio^†^

↑ Statistically significant increase.

↓ Statistically significant decrease.

↔ No statistical difference.

† IG overcomes comparator.

IG, Intervention Group; CG, Control Group; n.a. not available; RCT, Randomized Controlled Trial; MMAS, Modified Modified Ashworth Scale; TUG, Timed Up and Go; 10MWT, 10-m Walking Test; aROM, active Range of Motion; pROM, passive Range of Motion; PA, Pennation Angle; MT, Muscle Thickness; SLS, Single Leg Stance; BI, Barthel Index; FL, Fascicle Length; FMA, Fugl-Meyer Assessment; LoS, Limits of Stability test; MD, Maximal Displacement; PPT, Pressure Pain Thresholds; 2MWT, 2-m Walking Test.

Most studies worked on chronic patients that were administered a single dry-needling session. In only two studies this intervention was repeated more than once (43, 48). Four studies included a control group that was treated in one of three ways: by sham dry needling (the procedure consisted in applying blunt needles or by inserting the needle at predetermined non-acupoints); by a rehabilitation session according to Bobath's methodology, or with no intervention.

Five of the six studies saw positive changes in the primary outcome. Immediately after the dry-needling treatment, the MAS score decreased on average by 1 point. The two papers that reevaluated patients some weeks later recorded long-lasting improvements.

### Transcutaneous electrical nerve stimulation (TENS)

TENS has been tested in six studies and was applied on dorsiflexor and/or plantarflexor muscles with the aim of reducing TS spasticity causing EFD. The characteristics of these studies are illustrated in Table 5. Treatment delivery varied considerably: from 10 to 60 min sessions, with a 50–200 Hz frequency and 0.06–0.3 ms single stimulus duration. Half of the included studies analyzed the effects of TENS after a single treatment session, while the remaining offered 5 weekly interventions lasting 2–4 weeks. Treatments were delivered by physiotherapists in five studies and by MDs in one study.

**Table 5:** Characteristics of studies using Transcutaneous Electrical Nerve Stimulation (TENS) for the treatment of triceps spasticity after stroke.

**Author**	**Sample**	**Intervention and dosage**	**Comparator**	**Outcomes**	**Follow-up** **(months)**	**Within group** **difference (over** **the time)**	**Between group** **difference after** **treatment**
Koyama et al. (49)	20 patients with spasticity	30-min TENS (50/100/200 Hz) on the peroneal nerve (0.25 ms, 40 s ON/10 s OFF) One single session	n.a.	Reciprocal Inhibition (RI), Presynaptic Inhibition (PI) H-reflex latency	n.a.	PI ↑ RI, H-reflex ↔	n.a.
Picelli et al. (50) (RCT)	30 (10/10/10 IG1/IG2/IG3) patients with spasticity for at least 6 months (MAS>1)	IG1: 15-min TENS (100 Hz, 0.3 ms) on the tibialis nerve Five days a week, for 2 weeks	IG2: 10-min continuous ultrasounds on the triceps, 5 days a week for 2 weeks IG3: BoNT-A injections in the gastrocnemius	MAS pROM	3	IG1: MAS, pROM ↔ IG2: MAS, pROM ↔ IG3: MAS ↓ pROM ↑	MAS, pROM^§^ (IG3)
Cho et al. (51)	42 (22/20 IG/CG) patients with spasticity for at least 6 months	30-min Bobath rehabilitation + 60-min TENS (100 Hz, 0.2 ms) on the gastrocnemius One single session	CG: 30-min Bobath rehabilitation + sham TENS	MAS Plantarflexors resistance (PR) Postural sway length	One day	IG: MAS, PR, Postural Sway Length ↓ CG: MAS, HHD, Postural Sway Length ↓	MAS, PR, Postural sway length^†^ (but non at follow-up)
Martins et al. (52) (RCT)	20 patients with spasticity for at least 6 months (MAS ≥ 1)	IG1: 30-min TENS (100 Hz, 0.06 ms) on the posterior tibialis nerve One single session	IG2: 30-min cryotherapy (applied at the same group the following day) CG: 40 assessments of non-affected limbs before treatment	H/M ratio H-reflex latency, Tibialis anterior EMG activity	n.a.	IG1: H/M ratio ↓ H-reflex latency, Tibialis anterior EMG ↔ IG2: H/M ratio, H-reflex latency ↑ Tibialis anterior EMG ↔ CG: H/M ratio, H-reflex latency, Tibialis anterior EMG ↔	H/M ratio+ (CG) H-reflex latency^§^ (IG2)
Yan and Hui-Chan (53) (RCT)	56 (19/19/18 IG/CG1/CG2) patients with acute stroke (< 10 days)	60-min usual rehabilitation + 60-min TENS (100 Hz, 0.2 ms) on the acupuncture points of the lower limb Five days a week, for 3 weeks	CG1: 60-min usual rehabilitation + sham TENS CG2: 60-min usual rehabilitation only	Composite Spasticity Scale (CSS) MIVC of tibialis anterior and gastrocnemius, co-contraction of plantarflexors, TUG	2	IG: CSS, MIVC ↑ (CSS increased less than the other groups) TUG, co-contraction ↓ CG1: CSS, MIVC ↑ TUG, co-contraction ↓ CG2: CSS, MIVC ↑ TUG, co-contraction ↓	MIVC, TUG, co-contraction^†^
Ng and Hui-Chan (54) (RCT)	80 (19/21/20/20 IG1/IG2/CG1/CG2) patients with spasticity for at least 1 year (CSS ≥ 10)	IG1: 60-min TENS (100 Hz, 0.2 ms) IG2: 60-min TENS (100 Hz, 0.2 ms) + 60-min task-related training Five days a week, for 1 month	CG1: Sham TENS + 60-min task-related training CG2: No intervention	Composite Spasticity Scale (CSS) MIVC of dorsiflexors and plantarflexors, Gait velocity	1	IG1: CSS ↓ MIVC, velocity ↑ IG2: CSS ↓ MIVC, velocity ↑ CG1: CSS ↓ MIVC, velocity ↑ CG2: CSS, velocity ↔ MIVC ↑	CSS ^†^ (IG1, IG2) MIVC^†^ (IG2) Velocity^†^ (IG2)

↑ Statistically significant increase.

↓ Statistically significant decrease.

↔ No statistical difference.

† IG overcomes comparator (listed in brackets when multiple).

§ Comparator overcomes IG (listed in brackets when multiple).

IG, Intervention Group; CG, Control Group; n.a., not available; RCT, Randomized Controlled Trial; TENS, Transcutaneous Electrical Stimulation; PI, Presynaptic Inhibition; RI, Reciprocal Inhibition; MAS, Modified Ashworth Scale; BoNT-A, Botulinum Neurotoxin Type-A; pROM, passive Range of Motion; PR, Plantarflexors Resistance; EMG Electromyography; CSS, Composite Spasticity Scale; MIVC, Maximum Isometric Voluntary Contraction; TUG, Timed Up and Go.

MAS values were significantly reduced in a sample of chronic patients immediately after the single TENS session (mean difference of 1 point) compared to the sham stimulations (51), whereas in the RCT by Picelli et al. MAS did not improve at the one-month and three-month follow-ups (50). Two papers published by Yan and Ng with EFD patients with stroke-related spasticity reported that the score measured by the Composite Spasticity Scale decreased following TENS, even at the one-month follow-up (54). In acute patients TENS contributed in controlling the onset of spasticity more than in those doing rehabilitation alone (53). H-reflex measurements were only used by Martins et al. to assess muscle hyperreflexia. After a 30-min TENS session, H-reflex latency did not change, even though they reported a decrease in H/M ratio (52).

### Vibrations

In the treatment of EFD caused by TS spasticity, six studies used full body vibrations (see Table 6). Sessions lasted 5–10 min with the patient on a vibrating platform employing a 12–40 Hz frequency and a 1.5–4 mm amplitude. In four papers, the patient was standing on the platform with semi-flexed knees (55, 57, 59, 60), while in a study by Miyara the patient was sitting down (56, 58). Treatments were delivered by physiotherapists in five out of six studies.

**Table 6:** Characteristics of studies using vibrations for the treatment of triceps spasticity after stroke.

**Author**	**Sample**	**Intervention and dosage**	**Comparator**	**Outcomes**	**Follow-up** **(months)**	**Within group** **difference (over the** **time)**	**Between group** **difference after** **treatment**
Huang et al. (55) (RCT)	36 patients with spasticity for at least 6 months (MAS ≥ 1)	WBV (30 Hz, 1.5 mm amplitude), knees bent standing on a platform Five 1-min bouts, one single session	CG: Sham WBV on the same platform, 2 days later	H/M ratio Stiffness, Intramuscular blood perfusion (IBP)	n.a.	IG: H/M ratio ↓ IBP ↑ Stiffness ↔ CG: H/M ratio, stiffness, IBP ↔	H/M ratio, IBP^†^
Miyara et al. (56)	11 patients with spasticity for 3 months (MAS ≥ 1)	5-min WBV (30 Hz, 4–8 mm amplitude), seated + simultaneous triceps stretching One single session	Six healthy subjects	MAS aROM, pROM	n.a.	MAS ↓ aROM, pROM ↑	n.a.
Alp et al. (57) (RCT)	21 (10/11 IG/CG) patients with spasticity for at least 1 year (MAS ≥ 1)	Stretching + exercises + 5-min WBV (40 Hz, 4 mm amplitude), knees bent standing on a platform Three days a week, for 1 month	CG: Stretching + exercises + sham WBV on the same platform	MAS FIM, 10 mAS	One week, 1, 3, 6	IG: MAS, FIM ↔ 10 mAS (at 1 week, 1 and 3 months) ↑ CG: MAS, FIM, 10 mAS ↔	10 mAS^†^
Miyara et al. (58)	16 patients with spasticity (MAS ≥ 1)	5-min WBV (30Hz, 4 mm amplitude), seated One single session	n.a.	MAS F-waves, F/M ratio, aROM, pROM	n.a.	MAS, F-waves, F/M ratio ↓ aROM, pROM ↑	n.a.
Pang et al. (59) (RCT)	76 (38/39 IG/CG) patients with spasticity for at least 6 months	WBV (20–30 Hz, 0.44–0.6 mm amplitude), standing on a platform while performing specific exercises Three days a week, for 2 months	CG: Sham WBV on the same platform while performing specific exercises	MAS Chedoke McMaster Stroke Assessment (CMSA), Muscle power	1	IG: MAS, CMSA ↔ Muscle power ↑ CG: MAS, CMSA, Muscle power ↔	No statistical difference
Chan et al. (60) (RCT)	30 (15/15 IG/CG) patients with spasticity for at least 6 months (MAS ≥ 2)	WBV (12 Hz, 4 mm amplitude), knees bent standing on a platform Two 10-min bouts, one single session	CG: Sham WBV on the same platform	MAS Achilles Tendon reflex (ATR) excitability, H-reflex, H/M ratio, VAS, TUG, 10 mAS, Static Foot Contact Area (SFCA)	n.a.	IG: MAS, VAS, H/M ratio, TUG ↓ 10 mAS, SFCA ↑ ATR excitability, H-reflex ↔ CG: MAS, VAS, H/M ratio, TUG, 10mAS, SFCA, ATR excitability, H-reflex ↔	MAS, VAS, TUG, 10 mAS, SFCA^†^

↑ Statistically significant increase.

↓ Statistically significant decrease.

↔ No statistical difference.

† IG overcomes comparator.

IG, Intervention Group; CG, Control Group; n.a, not available; RCT, Randomized Controlled Trial; MAS, Modified Ashworth Scale; WBV, Whole Body Vibration; IBP, Intramuscular Blood Perfusion; FIM, Functional Independence Measurement; 10mAS, 10-meter Ambulation Scale; aROM, active Range of Motion; pROM, passive Range of Motion; CMSA, Chedoke McMaster Stroke Assessment; VAS, Visual Analog Scale; TUG, Timed Up and Go; SFCA, Static Foot Contact Area; ATR, Achilles Tendon Reflex.

The length of a treatment varied considerably among studies: in four studies, patients underwent a single session of vibration therapy (55, 56, 58, 60); in another study, Alp et al. devised a month-long program, lasting 12 sessions (57), and Pang's group vibration therapy lasted 2 months (59). Four of these studies were RCTs, which included sham vibrations control groups sometimes associated with specific exercises.

Five studies employed MAS to assess spasticity. There was an improvement immediately after treatment in three out of five studies. In the remaining two studies spasticity did not change after treatment, nor did it change in the long term (57, 59). The H/M ratio was used as the primary outcome in one RCT and improved after one session of vibration therapy (55).

### Ultrasounds

Ultrasound therapy is used to heat the underlying soft tissues. Ultrasounds were used to treat EFD with stroke-related spasticity in 5 RCTs. These studies are described in Table 7. Treatments were delivered by physiotherapists in four out of five studies.

**Table 7:** Characteristics of studies using ultrasounds for the treatment of triceps spasticity after stroke.

**Author**	**Sample**	**Intervention and dosage**	**Comparator**	**Outcomes**	**Follow-up** **(months)**	**Within groups** **difference (over the** **time)**	**Between group** **difference after** **treatment**
Radinmehr et al. (28) (RCT)	32 (16/16 IG1/IG2) patients with chronic spasticity (MMAS > 1)	IG1: 10-min continuous ultrasounds (1 MHz, 1.5 W/cm^2^) on the gastrocnemius One single session	IG2: Radial shock-wave therapy	MMAS aROM, pROM, TUG, H-reflex latency, PPFT	n.a.	IG1: MMAS, TUG, PPFT ↓ aROM, pROM, H-reflex latency ↑ IG2: MMAS, TUG, PPFT ↓ aROM, pROM, H-reflex latency ↑	No statistical difference
Picelli et al. (50) (RCT)	30 (10/10/10 IG1/IG2/IG3) patients with spasticity for at least 6 months (MAS>1)	IG1: 10-min continuous ultrasounds (1 MHz, 1.5 W/cm^2^) on the triceps Five days a week, for 2 weeks	IG2: 15-min TENS (100 Hz, 0.3 ms) on the tibial nerve, 5 days a week for 2 weeks IG3: BoNT-A injections in the gastrocnemius	MAS pROM	3	IG1: MAS, pROM ↔ IG2: MAS, pROM ↔ IG3: MAS ↓ pROM ↑	MAS, pROM^§^ (IG3)
Sahin et al. (61) (RCT)	41 (20/21 IG/CG) patients with spasticity for at least 1 year (MAS > 1)	Stretching + 10-min continuous ultrasounds (1.5 W/cm^2^) Five days a week, for 1 month	CG: Stretching 5 days a week, for 1 month	MAS aROM, pROM, H/M ratio, BMRS, FIM	n.a.	IG: MAS ↓ aROM, pROM ↑ H/M ratio, BMRS, FIM ↔ CG: MAS ↓ aROM, pROM ↑ H/M ratio, BMRS, FIM ↔	No statistical difference
Ansari et al. (62) (RCT)	21 (11/10 IG1/IG2) patients with spasticity (85% due to stroke)	IG1: 10-min continuous ultrasounds (1 MHz, 1.5 W/cm^2^) on the triceps One single session	IG2: 20-min infrared (500 W), one single session	MAS H/M ratio, aROM, pROM	n.a.	IG1: MAS, H/M ratio, aROM, pROM ↔ IG2: MAS, H/M ratio, aROM, pROM ↔	No statistical difference
Ansari et al. (63) (RCT)	12 (6/6 IG/CG) patients with spasticity for at least 6 months	10-min continuous ultrasounds (1 MHz, 1.5 W/cm^2^) on the triceps Three days a week, for 5 weeks	CG: Sham ultrasounds on the triceps, three days a week for 5 weeks	MAS H/M ratio, aROM, pROM	n.a.	IG: MAS, H/M ratio ↓ aROM, pROM ↔ CG: MAS, H/M ratio, aROM, pROM ↔	H/M ratio^†^

↑ Statistically significant increase.

↓ Statistically significant decrease.

↔ No statistical difference.

† IG overcomes comparator.

§ Comparator overcomes IG (listed in brackets when multiple).

IG, Intervention Group; CG, Control Group; n.a, not available; RCT Randomized Controlled Trial; MMAS, Modified Modified Ashworth Scale; aROM, active Range of Motion; pROM, passive Range of Motion; TUG, Timed Up and Go; PPFT, Passive Plantarflexor Torque; MAS, Modified Ashworth Scale; BoNT-A, Botulinum Neurotoxin Type-A; BMRS, Brunnstrom Motor Recovery Stage; FIM, Functional Independence Measurement.

The same parameters were used by all the authors and were set at 1 Hz, 1.5 W/cm^2^, lasting 10 min. Radinmehr and Ansari planned a single treatment session (28, 62), whereas in other studies the intervention lasted 2–5 weeks, with 3–5 weekly sessions. All RCTs included a control group suffering from EDF and TS spasticity. The control group underwent other types of physical or manual PT (shock waves, TENS, infrared therapy, stretching), placebo ultrasound sessions, or botulinum toxin injections.

Within-group MAS value improvements were found in three of the five studies. MAS decreased on average by 1 point. However, Ansari and Picelli did not record any substantial changes in the experimental group compared to the baseline values (50, 62). In the intervention groups some studies recorded long-lasting improvements in ankle ROM measurements, but no study found any statistical reduction when compared to the control groups. H-reflex-related outcomes were employed in four studies even though only two improved after an ultrasound treatment (28, 63). However, only the sample in the study by Ansari showed a marked difference compared to the sham ultrasound group (63).

Ultimately, ultrasounds overall were just as effective as other types of therapy. In fact, spasticity improved just as much with other treatments such as shock waves, stretching, infrared, botulinum toxin, and TENS therapies.

### Cryotherapy

Cryotherapy uses ice or other refrigerating substances to cool down the treated area. Three studies were conducted to test the efficacy of this treatment in the reduction of EFD with triceps spasticity. Paper characteristics are presented in Table 8. Treatments were delivered by physiotherapists in all the included studies.

**Table 8:** Characteristics of studies using cryotherapy for the treatment of triceps spasticity after stroke.

**Author**	**Sample**	**Intervention and dosage**	**Comparator**	**Outcomes**	**Follow-up** **(months)**	**Within group** **difference (over time)**	**Between group** **difference after** **treatment**
Alcantara et al. (64) (RCT)	16 (7/9 IG/CG) patients with spasticity for at least 6 months (MAS ≥ 1)	20-min cryotherapy (1 kg ice pack) One single session	CG: 1 kg pack filled with sand at room temperature	MAS Dorsiflexors and plantarflexors strength, gait analysis	n.a.	IG: MAS ↓ Strength, gait parameters ↔ CG: MAS, strength, gait parameters ↔	MAS^†^
Garcia et al. (65) (RCT)	16 (7/9 IG/CG) patients with spasticity for at least 6 months (MAS ≥ 1)	20-min cryotherapy (1 kg ice pack) One single session	CG: 1 kg pack filled with sand at room temperature	MAS Joint position sense	n.a.	IG: MAS ↓ Joint position sense ↔ CG: MAS, Joint position sense ↔	MAS^†^
Martins et al. (52) (RCT)	20 patients with spasticity for at least 6 months (MAS ≥ 1)	IG1: 30-min cryotherapy (delivered to the same group on the second day) One single session	IG2: 30-min TENS (100 Hz, 0.06 ms) in the posterior tibialis nerve (applied during the first day) CG: 40 assessments of non-affected limbs before treatment	H/M ratio H-reflex latency, Tibialis anterior EMG activity	n.a.	IG1: H/M ratio, H-reflex latency ↑ Tibialis anterior EMG ↔ IG2: H/M ratio ↓ H-reflex latency, Tibialis anterior EMG ↔ CG: H/M ratio, H-reflex latency, Tibialis anterior EMG ↔	H/M ratio^†^ (CG) H-reflex latency^†^ (CG, IG2)

↑ Statistically significant increase.

↓ Statistically significant decrease.

↔ No statistical difference.

† IG overcomes comparator (listed in brackets when multiple).

IG, Intervention Group; CG, Control Group; n.a, not available; RCT, Randomized Controlled Trial; MAS, Modified Ashworth Scale; pROM, passive Range of Motion; 6MWT, 6-meter Walking Test.

Patients included in the two studies by Alcantara and Garcia presenting chronic spasticity were treated by applying an ice pack on the calf for 20 min, while the control group was given a 1 kg, room temperature sand pack to mimic the pressure the ice pack exerts on the muscles. Immediately after one session of cryotherapy, MAS values in the intervention group reduced in both studies compared to the controls (see Table 8): from a median value of 1+ (range 1–2) to a median value of 1 (range 0–1+), and from a mean value (std) of 2.29 (0.49) to 1.29 (0.76) (64, 65). In the study by Martins et al., a group was treated once by applying TENS to the tibialis nerve, and the following day the same patients received 30 min of cryotherapy. Immediately after the application, the H/M ratio increased with cryotherapy (compared to TENS), exacerbating reflex responses (52). No study investigated the effects of cryotherapy over time.

### Physiotherapist-guided physical exercise

We considered as physiotherapist-guided physical exercise all those interventions that did not involve instrumental therapies or passive maneuvers (e.g., stretching) that were performed manually by health professionals. Two studies met these criteria and were included in the scoping review, as illustrated in Table 9.

**Table 9:** Characteristics of studies providing PT-guided physical exercise interventions for the treatment of triceps spasticity after stroke.

**Author**	**Sample**	**Intervention and dosage**	**Comparator**	**Outcomes**	**Follow-up** **(months)**	**Within group** **difference (over** **time)**	**Between group** **difference after** **treatment**
Zhang et al. (66) (RCT)	36 (18/18 IG/CG) patients with spasticity for at least 6 months (MAS ≤ 2)	40-min water-based exercises Five days a week, for 2 months	CG: 40-min land-based exercises	MAS MVIC of dorsiflexors and plantarflexors, FAC, Barthel Index (BI)	n.a.	IG: MAS ↔ MVIC, FAC, BI ↑ CG: MAS ↔ MVIC dorsiflexors, FAC, BI ↑	FAC, BI^†^
Akbari et al. (67)	34 (17/17 IG/CG) patients with spasticity for at least 1 year	Strengthening, balance, and functional exercises Three days a week, for 1 month	CG: Balance and functional exercises	MAS Strength	n.a.	IG: MAS ↓ Strength ↑ CG: MAS ↓ Strength ↑	MAS^†^

↑ Statistically significant increase.

↓ Statistically significant decrease.

↔ No statistical difference.

† IG overcomes comparator.

IG, Intervention Group; CG, Control Group; n.a, not available; RCT Randomized Controlled Trial; MAS, Modified Ashworth Scale; MVIC, Maximum Voluntary Isometric Contraction; FAC, Functional Ambulatory Category; BI, Barthel Index.

Zhang et al. conducted an RCT with patients performing water-based exercises lasting 2 months, while a control group did floor exercises for the same amount of time. At the end of the intervention, the MAS score did not change either within or between groups, while muscle strength and functional scales of ambulatory function did (66). In a 12-month clinical trial, Akbari et al. treated 34 patients suffering from stroke sequelae with 12 sessions of balance and functional exercises. The intervention group (17/34 patients) also followed an additional protocol with strengthening exercises. After 1 month, MAS score was reduced in both groups (see Table 9), with a statistically significant difference between groups, even though it was more pronounced in the intervention group (67).

## Discussion

To our knowledge, this is the first scoping review that methodically summarized all the available primary evidence specific to PT treatments for EFD in post-stroke patients. Scoping reviews are used for mapping literature surrounding such a broad topic, highlighting any gaps that need to be filled and suggesting what research should be done in future studies (13). For this reason, we collected all relevant characteristics of the interventions delivered to stroke patients to counteract EFD.

In order to be considered eligible for the current review, studies had to deliver at least a single PT intervention, aiming to reduce EFD and involving at least one spasticity outcome measure. Since we were interested in exploring PT interventions in terms of dosage and frequency, only primary studies were included, while secondary literature summarizing many results in a single value was not considered.

### Study design and rationale

More than half of the included studies (32 out of 53) were RCTs, and this design was more frequent in studies using devices (e.g., shock waves) rather than in studies using manual therapies (e.g., stretching). This may depend on the fact that device-based treatments are easier to include in controlled trials when delivering sham treatments. Moreover, RCTs are often performed when introducing innovative treatments. Conversely, manual therapy has been used for many years. Overall there has been a steady and welcome increase of RCTs over time. Out of the 32 studies, 25 were published in the last decade (2012–21) and 17 of these just during the last 5 years (2016–21).

Among interventions, stretching was analyzed in 12 studies, shock waves in 9 studies, electrical stimulation in 7, dry needling in 6 studies, TENS in 6 studies, vibration therapy in 6 studies, ultrasounds in 5, cryotherapy in 3, and active physiotherapy in 2 studies. Such a wide choice is closely related to the several different phenomena underlying EFD (1). The loss in muscle length and extensibility and the overall soft tissue changes (stiffness and contracture) can be targeted by stretching, shock waves, ultrasound, dry needling, and active physiotherapy. TENS, vibrations, and cryotherapy reduce muscle excitability by targeting neurophysiological mechanisms, such as the pre-synaptic inhibition and neuromuscular spindles sensitivity (51, 52). Electrical stimulation and active physiotherapy base their rationale on the need to strengthen the agonist-antagonist muscle complex.

In line with the rationales of such different treatments, the outcome variables used to assess treatment efficacy ranged from pure pathophysiological indicators (H/M ratio) to functional measurements of overall joint stiffness during walking (torque-angle ratio).

### Between-group comparisons

Thirty-seven studies involved both an intervention and a control group. When comparing the results, 73% of the intervention group results were significantly superior to those of the control group (27 studies out of 37). However, the effect size computation for MAS score and ankle ROM revealed small changes in most of the studies. The physical therapies that proved to be the most effective were dry needling in 4 out of 4 trials, shock waves in 4 out of 5 trials, and electrical stimulation in 4 out of 5 RCTs. Ultrasounds were the technique with the fewest significant results in between-group comparisons, especially when the control group underwent other types of physical therapy. The main flaw in these studies is the small sample size, which was often limited to 15–20 patients. This implies a limited study power, and therefore a high probability of not detecting, when present, the efficacy of a treatment.

### Within-group comparisons

By analyzing spasticity also over the long run, an overall improvement within the intervention groups was found. MAS score, which was the main spasticity measurement used among all studies, showed an overall reduction by ~1 point (from 2 to 3 and from 1 to 2). Only Ng and Yan used the Composite Spasticity Scale to assess triceps spasticity and reported a significant decrease after TENS treatments (53, 54). Pradines' group used the Tardieu Angle to assess triceps spasticity and reported a significant decrease in spasticity after self-managed daily stretching sessions over a 1-year period (15, 17).

Passive ankle ROM was investigated in 25 studies. Joint degrees were measured using a handheld goniometer in 22 studies (88%) and by using mechanical devices in the remaining three. Among studies, ROM increased between 2 and 17.5 degrees toward dorsiflexion. The effect size for ROM improvement was medium or large in 16 out of 25 studies. Noteworthy, in a study by Santamato et al., where EFD was successfully treated after a single session of shock waves, bringing the ankle back to a neutral position (33).

In agreement with the results obtained from the between-group comparison, shock waves, dry needling, and electrostimulation were the interventions that, over time, had a greater impact in terms of spasticity in within-group analyses. Ultrasound therapy obtained the most mixed results when comparing baseline values.

In synthesis, at the end of each treatment many studies reported a statistically significant improvement in at least one of the outcomes. Nonetheless, this does not necessarily imply that this outcome is clinically significant. Indeed, minimal changes in MAS score (e.g., <1 point), or of a few more degrees in ankle dorsiflexion, do not determine functional improvements in patients who suffer from a severe form of EFD, such as to justify a choice of treatment over another. Similarly, only 6 studies recorded an increase in joint ROM ≥10 degrees, i.e., greater than the error of measurement when using handheld goniometers (~5 degrees).

As stated by the guidelines for scoping reviews (11, 13), we did not perform a statistical synthesis for treatment efficacy across studies, weighting them by sample size, as systematic reviews and meta-analyses do. We simply mapped what authors reported. No inference on the overall efficacy of physiotherapy on EFD can be done with such a study design. Instead, our findings outline several factors to take into consideration when designing a meta-analysis, including the type of intervention, frequency, duration, and activity of the control group.

### Time from the stroke: Acuteness and chronicity

The time elapsed between the onset of the stroke and the start of the intervention is of paramount importance when analyzing treatment efficacy, because of the progressive worsening of muscle shortening and soft tissue rheological modifications. Of the selected studies, 29 set the presence of chronicity as an inclusion criterion, 10 did not establish a time limitation from the onset of the acute event, and 13 included patients in the subacute phase (1–5 months). To date, only one study investigated patients in the acute phase. The first few weeks after a stroke have been proven to be a critical period for neuronal plasticity (68) and muscle rearrangement alike (69). Therefore, new studies should start to focus on the acute phase by testing whether specific treatments and dosages can alleviate stroke consequences in terms of spasticity and loss of joint ROM. For example, a recent RCT study tested the effects of a daily stretching protocol on a sample of 60 acute hospitalized patients, comparing it to the usual care provided in the same ward (70). After 1 month, the ankle ROM measurements demonstrated the effectiveness of the treatment in preventing the development of EFD, since dorsiflexion was preserved more in the intervention group than in the untreated group. However, this study was not included in the current review as the full text was not available (70). When dealing with chronic deformities, the self-care strategy put forward by Pradines (15) deserves to be further developed and investigated, given the significant improvements obtained at both the structural and functional levels at the 1-year mark and its lower impact, in terms of costs, on the national health systems.

### The dosage issue

When dealing with chronic deformities, knowing if a therapy is feasible and how long it must last before obtaining meaningful results is a fundamental factor. Under the umbrella term “dosage,” we considered the duration of the experimental treatment, the weekly frequency, how long a session lasted, and for instrumental therapies, the setting parameters used by each study. More than half of the studies (28 in total) investigated the effects of a single therapy session, while 15 studies recommended interventions lasting 1–4 weeks, and only 10 studies used longer treatments, including Pradines' 2 year-long papers (15, 17) on self-administered stretching exercises. There seems to be a consensus on the duration of the single sessions and instrumental parameters for stretching, shock waves, TENS, vibrations, ultrasounds, and cryotherapy. In contrast, electrical stimulation is still set up in very different ways among studies, as are physiotherapist-guided exercises. Comparators were also heterogeneous among studies, indicating a need for greater standardization. Scientific Societies could encourage discussions on these topics, even promoting the use of evidence-based methodologies in order to reach an agreement among experts, such as the Delphi Panels and the Consensus Conferences (71, 72).

### Future implications

New clinical intervention studies on PT interventions for EFD reduction could benefit from the topics highlighted in this scoping review. In the current study, we pointed out the variety of the rehabilitative approaches provided in literature and the dosage of each individual study. This heterogeneity is in line with recent literature surrounding spasticity, which is a very complex phenomenon, characterized by different alterations of central and peripheral origin that co-exist and influence one another (73, 74). The umbrella term “spasticity” should therefore be broken down, and clinicians should distinguish for each patient the role of the reflex components (e.g., spasticity, co-contraction, spastic dystonia) and passive components (muscle stiffness, viscosity, contractures, along with non-muscular tissue alterations) through appropriate assessment tools (7, 75). Future studies should clarify which is the target phenomenon of their study among those listed above, and be consistent in choosing the appropriate experimental treatment, according to its rationale (76). Stretching, shock waves, ultrasound, and dry needling mainly determine changes in soft tissue morphology and should be utilized if the primary cause for an increased response to stretching is peripheral. On the other hand, TENS, vibrations, electrical stimulation, and cryotherapy mostly affect central mechanisms, and should be therefore employed when targeting reflex arc-related phenomena. If a more appropriate taxonomy were used, specific scales or devices should be adopted to assess each of the abovementioned components. Currently, most RCTs use the MAS score to assess the efficacy of treatments. The main drawback of this approach is that it is not possible to recognize which reflex or passive components benefits from the treatment. Moreover, only after identifying which “spasticity” component contributes to movement alteration, it will be possible to optimize physiotherapy treatments and use only those therapies that affect a specific issue. The MTS is a clinical scale that distinguishes the stretching response after two stretching maneuvers performed at different speeds. If there is a difference between the two passive maneuvers, part of the resistance is due to a velocity-related central phenomena. Many neurophysiological measures, such as the H/M ratio or the H-reflex, can provide direct information on neural characteristics. Gait analysis with dynamic EMG performed while walking and during functional tasks can produce more in-depth results surrounding central and peripheral interactions (1, 77). Authors should always provide dosage parameters to allow studies to be replicated and be comparable in quantitative synthesis, including suitable control groups.

As there are still few PT treatments performed in the acute phase, future studies should focus on the immediate phase after a stroke, so as to identify treatments that could prevent or limit the development of EFD. Likewise, there is also a need to create a strong body of evidence on chronic management of TS spasticity over the long term, through the empowerment of the patients and the development of self-management skills, to avoid the onset of chronic deformities and without having to always refer back to public health institutions. For this reason, authors should also consider the inclusions of follow-up evaluations assessing the maintenance of long-term effects of PT interventions.

In this review we mainly focused on the outcome measurements of body structures, as per the ICF model. Therefore, measures used that pertain to the abilities and performance domains were only reported in tables. The choice of the most appropriate outcome measure and subsequent management of resulting data with proper statistical analysis are topics of extreme relevance. Due to their complexity, these deserve separate in-depth scrutiny, and future studies should focus on these issues.

It is widely known that segmental spasticity does not always correlate with functional movement (1, 78). In fact, spasticity is velocity-dependent, and stroke patients often have significantly reduced walking speed (79). Stretching speed achieved at the bedside during passive maneuvers, which is capable of eliciting overactivity, may not be accomplished during functional movement (1).

Meanwhile, patients may not be able to transfer segmental motor control competences acquired after PT treatments to their functional daily activities, since the latter require more complex skills.

In this review, as many as 32 out of the 53 studies included measures related to functional movement such as standing or walking, like the 10-meter Walking Test, the Timed Up&Go, and the Berg Balance Scale (see Tables 1–9). Only 16 studies reported a significant improvement in functional outcomes. In future studies, measures of segmental spasticity should always be performed alongside measures of functional ability. Moreover, dynamic instrumental measures should also be considered, thus allowing to distinguish the underlying causes of altered gait patterns (75). Only by combining all of the above-mentioned measures, the phenomenon of overactivity could be understood thoroughly in all its aspects and properly assess the efficacy of PT treatments on functional activities.

This scoping review aimed to map existing literature on PT treatment for EFD due to TS spasticity in stroke patients. According to specific PRISMA guidelines (12), we did not perform a critical appraisal of the included studies since no quantitative synthesis nor meta-analysis was conducted. This review does not intend to give readers specific answers about the real effectiveness of PT treatments, but rather to provide an overview on the available scientific literature, discussing the gaps of knowledge that need to be addressed by future studies.

Moreover, unlike systematic reviews, the scoping review framework does not require these protocols to be published on dedicated repositories beforehand. For this reason, a preliminary peer-review process on the methodology is missing. This represents the main limitation of our study. The string search was created following an iterative process aimed at improving the sensibility of the search to compensate precisely for this limitation, as suggested by scoping reviews guidelines. Nonetheless, some articles may still have been overlooked during the database search.

## Conclusions

This review collected all available studies on PT-deliverable treatments for EFD.

Our work provides useful insights for professionals working with stroke patients, who can select the most appropriate treatment according to the component mainly responsible for the deformity in a specific patient.

This scoping review also highlighted the interventions' main characteristics that researchers should consider when designing either a new clinical trial or a systematic review to produce a quantitative synthesis with clinical and practical implications.

## Data availability statement

The original contributions presented in the study are included in the article/Supplementary material, further inquiries can be directed to the corresponding author/s.

## Author contributions

Conceptualization: IC and AM. Data curation: IC, MBò, FS, and AM. Formal analysis: IC, MBò, and AM. Methodology and writing—original draft: IC, MBa, and AM. Supervision: IC, BD, SS, ML, and AM. Writing—review and editing: IC, MBò, FS, MBa, BD, SS, ML, and AM. All authors contributed to the article and approved the submitted version.

## Funding

This study was entirely funded by the Azienda USL-IRCCS of Reggio Emilia.

## Conflict of interest

Authors MBò and AM were also employed by Merlo Bioengineering. The remaining authors declare that the research was conducted in the absence of any commercial or financial relationships that could be construed as a potential conflict of interest.

## Publisher's note

All claims expressed in this article are solely those of the authors and do not necessarily represent those of their affiliated organizations, or those of the publisher, the editors and the reviewers. Any product that may be evaluated in this article, or claim that may be made by its manufacturer, is not guaranteed or endorsed by the publisher.
