# Supplementary material for: Physical therapy interventions for the correction of equinus foot deformity in post-stroke patients with triceps spasticity: A scoping review
Source: Front Neurol. 2022 Oct 28;13:1026850. doi: 10.3389/fneur.2022.1026850 (PMC9650131; doi:10.3389/fneur.2022.1026850)
Supplement: Supplementary file 1 [file Table_1.DOCX]

Supplementary Material

Physical Therapy Interventions for the Correction of Equinus Foot Deformity in Post-Stroke Patients with Triceps Spasticity: A Scoping Review

Isabella Campanini^1*^, Maria Chiara Bò^1,2^, Francesca Salsi^1^, Maria Chiara Bassi^3^, Benedetta Damiano^1^, Sara Scaltriti^1^, Mirco Lusuardi^4^, Andrea Merlo^1,2^

# Supplementary Figures and Tables

| **Database** | **Search strategy** | **Notes** |
| --- | --- | --- |
| **Medline (Pubmed)** | (triceps surae OR triceps OR gastrocnem* OR soleus OR "Equinus Deformity"[Mesh] OR equinus foot OR equinus deformity OR equinovarus foot OR equinovarus deformity) AND ("Stroke"[Mesh] OR Acute Stroke* OR Acute Cerebrovascular Accident* OR subacute Stroke OR "cerebrovascular disorders" [MeSH] OR "brain ischemia" [MeSH] OR "intracranial hemorrhages" [MeSH] OR "brain infarction" [MeSH] OR poststroke OR post-stroke OR cerebrovasc* OR cerebral* OR ischemi* OR haemorr*) AND (rehabilitation OR physiotherapy OR stretching OR shock-wav* OR "High-Energy Shock Waves"[Mesh] OR "Extracorporeal Shockwave Therapy"[Mesh] OR dry needling OR "Dry Needling"[Mesh] OR "Muscle Stretching Exercises"[Mesh] OR passive mobilization OR "Ultrasonic Waves"[Mesh] OR ultrasound* OR vibration therapy OR vibration* OR "Transcutaneous Electric Nerve Stimulation"[Mesh] OR tens OR "Electric Stimulation Therapy"[Mesh] OR electric stimulation) AND ("Muscle Spasticity"[Mesh] OR "Muscle Hypertonia"[Mesh] OR muscle spasticity OR muscle hypertonia OR spasticity OR spastic paresis OR overactivity OR spastic myopathy) | Search filters:   - Adult: 19+ years |
| **Cinahl** | (MH "Equinus Deformity" OR triceps surae OR triceps OR gastrocnem* OR soleus OR equinus foot OR equinus deformity OR equinovarus foot OR equinovarus deformity ) AND (MH "Cerebral Infarction" OR MH "Stroke" OR MH "Cerebrovascular Disorders" OR MH "Intracranial Hemorrhage" OR MH "Hypoxia-Ischemia, Brain" OR Acute Stroke* OR Acute Cerebrovascular Accident* OR subacute Stroke OR poststroke OR post-stroke OR cerebrovasc* OR cerebral* OR ischemi* OR haemorr* OR stroke) AND (Rehabilitation OR physiotherapy OR stretching OR shock-wav* OR dry needling OR passive mobilization OR ultrasound* OR vibration therapy OR vibration* OR tens OR electric stimulation or MH "Electric Stimulation" OR MH "Transcutaneous Electric Nerve Stimulation" OR MH "Stretching" OR MH "Dry Needling") AND (muscle spasticity OR muscle hypertonia OR spasticity OR spastic paresis OR overactivity OR spastic myopathy OR MH "Muscle Hypertonia" OR MH "Muscle Spasticity" ) |  |
| **Cochrane database** | (triceps surae OR triceps OR gastrocnem* OR soleus OR equinus foot OR equinus deformity OR equinovarus foot OR equinovarus deformity) in Title Abstract Keyword AND (Stroke OR Acute Stroke* OR Acute Cerebrovascular Accident* OR subacute Stroke OR intracranial hemorrhages OR brain infarction OR poststroke OR post-stroke OR cerebrovasc* OR cerebral* OR ischemi* OR haemorr*) in Title Abstract Keyword AND (rehabilitation OR physiotherapy OR stretching OR shock-wav* OR dry needling OR passive mobilization OR ultrasound* OR vibration therapy OR vibration* OR tens OR electric stimulation) in Title Abstract Keyword AND (muscle spasticity OR muscle hypertonia OR spasticity OR spastic paresis OR overactivity OR spastic myopathy) in Title Abstract Keyword |  |
